# Predictive Ability of C-Reactive Protein in Detecting Short-Term Complications After Cytoreductive Surgery and Hyperthermic Intraperitoneal Chemotherapy: A Retrospective Cross-Sectional Study

**DOI:** 10.1245/s10434-020-08619-y

**Published:** 2020-06-10

**Authors:** Job P. van Kooten, Arvind Oemrawsingh, Nadine L. de Boer, Cornelis Verhoef, Jacobus W. A. Burger, Eva V. E. Madsen, Alexandra R. M. Brandt-Kerkhof

**Affiliations:** 1 Department of Surgical Oncology, Erasmus MC Cancer Centre, Rotterdam, The Netherlands; 2 Department of Surgery, Catharina Hospital, Eindhoven, The Netherlands

## Abstract

**Background:**

Cytoreductive surgery (CRS) with hyperthermic intraperitoneal chemotherapy (HIPEC) is a potentially curative treatment for peritoneal carcinomatosis.

**Objective:**

The aim of this study was to determine the predictive value of postoperative inflammatory biomarkers in assessing complications after CRS and HIPEC.

**Methods:**

A prospective database of 181 patients, who underwent CRS-HIPEC between March 2014 through April 2018 in the Erasmus MC, was retrospectively analyzed. Postoperative complications were defined according to the serious adverse event (SAE) grading system. Levels of C-reactive protein (CRP) and white blood cell (WBC) count were compared between patients with SAE grade < 3 and SAE grade ≥ 3. The area under the receiver operating characteristic curve (AUC) was calculated for CRP and WBC against SAE ≥ 3 and various intra-abdominal complications.

**Results:**

SAE ≥ 3 postoperative complications occurred in 50 patients. From the second until the fifth postoperative day (POD), CRP levels were significantly higher (*p* = 0.023, *p* < 0.001, *p* = 0.002, and *p* = 0.002, respectively) in these patients. CRP concentrations above 166 mg/L on POD3 (AUC 0.75) and 116 mg/L on POD4 (AUC 0.70) were associated with the highest risk of an SAE ≥ 3. Postoperative WBC levels were not significantly different between patients with SAE < 3 and SAE ≥ 3 complications.

**Conclusion:**

Data from our hospital suggest that CRP levels that continue to rise after POD2 or that are ≥ 166 mg/L at POD3 or ≥ 116 mg/L at POD4, indicate a considerable risk for developing high-grade SAEs. The cut-off values we found can potentially be used as a threshold for additional diagnostic interventions, after they have been validated in external data.

Cytoreductive surgery combined with intraoperative hyperthermic intraperitoneal chemotherapy (CRS-HIPEC) has been considered a potentially curative therapeutic modality for patients presenting with peritoneal carcinomatosis (PC).^1^–^3^ This extensive surgical treatment has been associated with improved survival outcomes for selected patients with PC from colorectal cancer (CRC) and pseudomyxoma peritonei (PMP), with 5-year survival rates of approximately 30% and 74% for CRC and PMP, respectively.^4^–^6^ However, it has also been associated with considerable postoperative morbidity and mortality, with estimates of approximately 30% and 2–3%, respectively.^7^–^9^ When attempting to reduce postoperative morbidity and mortality, early recognition of high-grade serious adverse events (SAEs) could be of great significance.

C-reactive protein (CRP) is an acute-phase inflammation protein secreted primarily by liver hepatocytes, smooth muscle cells, and adipocytes, among others.^10^ With its half-life being only 19 h and its increase being proportional to the degree of the inflammation process, CRP has established itself as an inexpensive, highly sensitive but non-specific biomarker of systemic inflammatory response,^11^,^12^ and has been identified as a potential predictive marker of postoperative complications after abdominal surgery.^13^,^14^ Intra-abdominal complications, mainly septic complications or anastomotic leakage, are associated with mortality, reoperation, increased hospital stay, and higher costs.^15^

Research on the utility and predictive value of biomarkers, such as CRP and WBC (white blood cell) levels, after CRS and HIPEC has been limited.^16^ The aim of this cross-sectional retrospective study was to determine the predictive value of postoperative CRP and WBC levels in identifying complications after CRS and HIPEC in patients with PC from CRC or PMP.

## Methods

### Study Population

All patients with PC from CRC or PMP who underwent CRS-HIPEC in the Erasmus Medical Center between March 2014 through April 2018 were included in this study. A prospective database was built based on patients’ chart review by using the electronic medical record system at this institution. Patients with recurrent peritoneal disease who underwent a second CRS-HIPEC procedure in the aforementioned time interval, were also included.

### Perioperative Course

CRS-HIPEC procedures were performed by a specialized surgical team and in accordance with Dutch CRS and HIPEC protocols.^17^ After abdominal access via laparotomy, a thorough assessment of the extent of peritoneal disease (only in cases with PC from colorectal and appendiceal cancer) was conducted to determine the Peritoneal Cancer Index (PCI) score according to Jacquet and Sugarbaker.^18^ If the PCI score was under 20 and/or the specialized surgeons deemed the peritoneal disease resectable, the greater omentum, primary tumor (if still present), affected visceral abdominal organs, affected parietal surfaces, and all peritoneal implants were resected. Administration of HIPEC was by way of the open (coliseum) technique in which the abdominal cavity was filled with an iso-osmotic glucose/electrolyte dialysis (Dianeal^®^) carrier solution, with either mitomycin-C or oxaliplatin being added to the perfusate as chemotherapeutic agent, once the desired abdominal temperature of > 40 °C was reached. After the HIPEC perfusion, intestinal bowel anastomoses and/or a stomy procedure was performed if necessary.

Postoperatively, patients were treated following standard of care for CRS-HIPEC procedures. Laboratory tests and diagnostic imaging modalities, such as computed tomography (CT) scans, were liberally used when deemed necessary. Postoperative complications were retrospectively classified according to the SAE grading system: SAE = 1 denotes an asymptomatic or mild complication (intervention not indicated); SAE = 2 denotes a moderate complication (local or non-invasive intervention indicated); SAE = 3 denotes a severe complication (significant but not immediately life-threatening, radiological or surgical intervention indicated); SAE = 4 denotes a life-threatening complication (reoperation and/or prolonged intensive care unit [ICU] stay indicated); and SAE = 5 denotes in-hospital death related to the adverse event.^17^,^19^ Intra-abdominal gastrointestinal complications included anastomotic leakage, bowel perforation or ischemia/necrosis. The postoperative period was defined as the duration of the entire hospital stay following CRS and HIPEC, regardless of length.

### Laboratory Data

Laboratory results (including postoperative biomarkers) of all patients who underwent CRS-HIPEC were recorded on arrival to the ICU and then daily during the patient’s usually brief stay (1–3 days). When transferred to the ward, CRP levels were drawn in addition to a complete blood count (CBC), including white blood cell (WBC) count and blood chemistry in patients, usually three times a week (according to the Erasmus MC CRS-HIPEC protocol). CRP and WBC levels were routinely measured on postoperative days (PODs) 1, 2, 3, 4, and/or 5. Laboratory data were gathered retrospectively.

### Statistical Analysis

Quantitative variables are presented as median with interquartile range (IQR), while categorical variables are presented as counts with percentages. Daily postoperative CRP values and WBC count between the SAE < 3 and SAE ≥ 3 groups were compared using the Mann–Whitney U-test (non-parametric). All tests were performed two-sided and results were considered significantly different when the *p* value was < 0.05. Diagnostic accuracy of CRP and WBC values on consecutive PODs was analyzed using the receiver operating characteristic (ROC) curve by calculating separate cut-off levels for CRP and WBC with optimal sensitivity and specificity. Outcomes assessed were intra-abdominal gastrointestinal complications, intra-abdominal abscess, and SAE ≥ 3. Areas under the receiver operating characteristic curves (AUCs) were used to compare ROC curves. Statistical analyses were performed using the Statistical Package for Social Sciences (SPSS) version 21.0 (IBM Corporation, Armonk, NY, USA).

### Ethical Considerations

All study procedures were performed according to the Erasmus MC Research Codes and with permission of the local Medical Ethics Review Committee (MEC-2018-1286).

## Results

### Study Population

From March 2014 to May 2018, 181 patients underwent CRS-HIPEC in the Erasmus Medical Center. Patient- and tumor-related characteristics are described in Table 1. Three patients underwent a re-HIPEC within the aforementioned time interval, bringing the total number of analyzed CRS-HIPEC procedures to 184. Primary tumors included 147 colorectal adenocarcinomas (81.2%), 22 PMPs (12.2%), and 12 appendiceal adenocarcinomas (6.6%). Comparison of the baseline characteristics showed that the SAE ≥ 3 group comprised significantly more male patients (67% vs. 44%; *p* = 0.006). No further differences at baseline existed between groups.

**Table 1:** Baseline characteristics

	All patients [*N* = 181]	SAE < 3 [*n *= 133]	SAE ≥ 3 [*n *= 48]	*p* value
Female	91 (50.3)	75 (56.4)	16 (33.3)	0.006
Age, years	62 [53–69]	60 [52–70]	64 [56.3–68.8]	NS
BMI	25.6 [22.8–29]	25.4 [22.3–28.7]	26.4 [24.1–29.4]	NS
Smoking (past or current)	88 (48.6)	60 (45.1)	28 (58.3)	NS
Diabetes	21 (11.6)	14 (10.5)	7 (14.6)	NS
IDDM	8 (38)	6 (42.9)	2 (28.6)	NS
Hypertension	42 (23.2)	29 (21.8)	13 (27.1)	NS
ASA classification				NS
1	34 (18.8)	28 (21.1)	6 (12.5)	
2	115 (63.5)	84 (63.2)	31 (64.6)	
3	32 (17.7)	21 (15.8)	11 (22.9)	
Primary tumor				NS
Appendix cancer	12 (6.6)	8 (6)	4 (8.3)	
PMP	22 (12.2)	15 (11.3)	7 (14.6)	
CRC ascending colon	60 (33.1)	47 (35.3)	13 (27.1)	
CRC transverse colon	10 (5.5)	7 (5.3)	3 (6.3)	
CRC descending colon	16 (8.8)	12 (9.0)	4 (8.3)	
CRC sigmoid	39 (21.5)	29 (21.8)	10 (20.8)	
CRC rectum	22 (12.2)	15 (11.3)	7 (14.6)	
PC diagnosis				NS
Synchronous	88 (48.6)	65 (48.9)	23 (47.9)	
Metachronous	93 (51.4)	68 (51.1)	25 (52.1)	

Continuous variables are reported as median [IQR], and proportions are reported as *n* (%)

*SAE* serious adverse event, *BMI* body mass index, *IDDM* insulin-dependent diabetes mellitus, *ASA* America Society of Anesthesiologists, *PMP* pseudomyxoma peritonei, *PC* peritoneal carcinomatosis, *IQR* interquartile range, *NS* non-significant, *CRC* colorectal cancer

### Intraoperative and Postoperative Course

Table 2 reports the intraoperative course characteristics. Median procedure time of CRS-HIPEC was 398 min [327–475]. Of all patients with peritoneally disseminated CRC (*n* = 147), the median PCI was 10.^6^–^16^ Bowel anastomosis was performed in 109 procedures (59.2%), and median blood loss was 1183 mL [714–2075]. In the SAE ≥ 3 group, significantly more cholecystectomies were performed (16% vs. 3.7%; *p* = 0.004). In addition, median intraoperative blood loss was significantly more in the SAE ≥ 3 group (1533 mL [900–2700] vs. 1063 mL [688–1763]; *p* = 0.005). SAE score ≥ 3 complications occurred after 50 (27.2%) procedures (Table 3), of which 36% were gastrointestinal complications (anastomotic leakage and bowel perforation/ischemia). The most frequently occurring major complications in the cohort were intra-abdominal abscess (11.4%), anastomotic leakage (4.9%), intra-abdominal bleeding (3.8%), and pulmonary embolisms (3.8%). Reoperation had to be performed in 28 (15.2%) patients. Median duration of hospital stay was 17 days.^13^–^23^

**Table 2:** Intraoperative characteristics

	All procedures [*N* = 184]	SAE < 3 [*n* = 134]	SAE ≥ 3 [*n* = 50]	*p* value
PCI^a^	10 [6–16]	10 [5–16]	14 [8–16]	NS
(Partially) resected organs				
Omentum	174 (94.6)	128 (95.5)	46 (94)	NS
Spleen	12 (6.5)	6 (4.5)	6 (12)	NS
Urether	5 (2.7)	3 (2.2)	2 (4)	NS
Bladder	4 (2.2)	4 (3)	0	NS
Uterus^b^	36 (39.6)	28 (20.9)	8 (16)	NS
Ovaries^b^	63 (69.2)	48 (35.8)	14 (28)	NS
Stomach	2 (1.1)	1 (0.7)	1 (2)	NS
Liver	10 (5.4)	7 (5.2)	3 (6)	NS
Pancreas	11(6)	7 (5.2)	4 (8)	NS
Gallbladder	12 (6.5)	4 (3)	8 (16)	0.004
Duodenum	10 (5.4)	4 (3)	1 (2)	NS
Small intestine	52 (28.3)	35 (26.1)	17 (34)	NS
Appendix	10 (5.4)	7 (5.2)	3 (6)	NS
Ileocecal	22 (12)	13 (9.7)	9 (18)	NS
Ascending colon	40 (21.7)	27 (20.1)	13 (26)	NS
Transverse colon	21 (11.4)	12 (9)	9 (18)	NS
Descending colon	12 (6.5)	9 (6.7)	3 (6)	NS
Sigmoid colon	55 (29.9)	39 (29.1)	16 (32)	NS
Rectum	47 (25.5)	32 (23.9)	15 (30)	NS
Diaphragm				
Left	20 (10.9)	14 (10.4)	6 (12)	NS
Right	42 (22.8)	27 (20.1)	15 (30)	NS
Peritoneum				
Left	59 (32.1)	43 (32.1)	16 (32)	NS
Right	73 (39.7)	52 (38.8)	21 (42)	NS
Pelvic	71 (38.6)	57 (42.5)	14 (28)	NS
CCR score				NS
R1	177 (96.2)	130 (97)	47 (94)	
R2a	5 (2.7)	3 (2.2)	2 (4)	
R2b	2 (1.1)	2 (1.5)	0	
Number of bowel anastomoses				NS
0	75 (40.8)	60 (44.8)	15 (30)	
1	86 (46.7)	62 (46.3)	24 (48)	
2	17 (9.2)	8 (6)	9 (18)	
3	4 (2.2)	2 (1.5)	2 (4)	
4	2 (1.1)	2 (1.5)	0	
Stomy				NS
Ileostomy	11 (6)	7 (5.2)	4 (8)	
Colostomy	61 (33.2)	44 (32.8)	17 (34)	
Double barrel colostomy	2 (1.1)	2 (1.5)	0	
HIPEC regimen				NS
Mitomycin-C	164 (89.1)	121 (90.3)	43 (86)	
Oxaliplatin (5-fluorouracil, leucovorin)	11 (6)	9 (6.7)	2 (4)	
Other	9 (4.9)	5 (3.7)	4 (8)	
Blood loss, mL	1183 [714–2075]	1063 [688–1763]	1533 [900–2700]	0.005
Procedure time, min	398 [327–475]	396 [326–454]	399 [325–515]	NS

Continuous variables are reported as median [IQR], and proportions are reported as *n* (%)

*SAE* serious adverse event, *PCI* Peritoneal Cancer Index, *CCR* completeness of cytoreduction, *IQR* interquartile range, *NS* non-significant, *CRS*-*HIPEC* cytoreductive surgery-hyperthermic intraperitoneal chemotherapy

^a^Applicable to CRS-HIPEC procedures for colorectal cancer (*n *= 147)

^b^Proportion of CRS-HIPEC procedures in female patients (*n *= 91)

**Table 3:** Postoperative course

	All procedures [*N* = 184]	SAE < 3 [*n *= 134]	SAE ≥ 3 [*n *= 50]
Complications			
Intra-abdominal bleeding	7 (3.8)	1 (0.7)	6 (12)
Anastomotic leakage^a^	9 (4.9)	0	9 (18)
Bowel perforation/ischemia	9 (4.9)	0	9 (18)
Wound dehiscence	5 (2.7)	1 (0.7)	4 (8)
Intra-abdominal abscess	21 (11.4)	3 (2.2)	18 (36)
Wound infection	20 (10.9)	15 (11.2)	5 (10)
UTI	16 (8.7)	14 (10.4)	2 (4)
Pneumonia	8 (4.3)	6 (4.5)	2 (4)
Pulmonary embolism	7 (3.8)	2 (1.5)	5 (10)
SAE grade		–	–
0	58 (31.5)		
1	29 (15.8)		
2	46 (25)		
3	24 (13)		
4	20 (10.9)		
5	6 (3.3)		
Reoperation	28 (15.2)	0	28 (56)
ICU stay	3 [2–3]	3 [2–3]	4 [3–8]
Hospital stay	17 [13–23]	15.5 [12–18.3]	30 [19–39]
In-hospital mortality	6 (3.3)	0	6 (12)

Continuous variables are reported as median [IQR], and proportions are reported as *n* (%)

*SAE* serious adverse event, UTI urinary tract infection, *ICU* intensive care unit, *IQR* interquartile range, *CRS*-*HIPEC* cytoreductive surgery-hyperthermic intraperitoneal chemotherapy

^a^Proportion of all patients with bowel anastomosis after CRS-HIPEC (*n *= 109)

### Postoperative Biomarkers

**Fig. 1:**
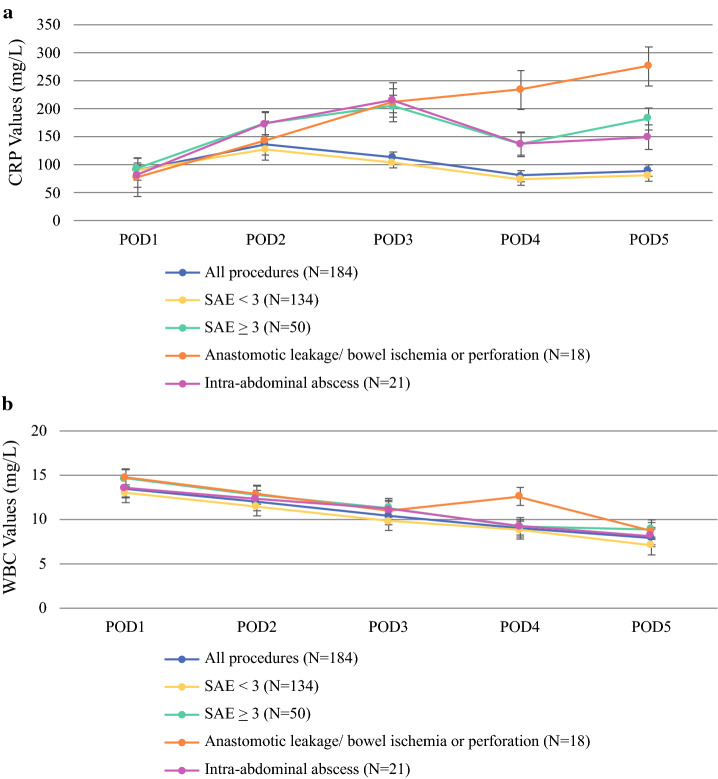
**a** Postoperative CRP values after CRS and HIPEC. **b** Postoperative WBC count after CRS and HIPEC. *POD* postoperative day, *CRP* C-reactive protein, *SAE* serious adverse event, *WBC* white blood cell count

The overall evolution of median CRP values up to POD 5 is shown in Fig. 1a. Overall, the median CRP value increased from 89.5 mg/L [67.3–126] (POD 1) to a peak of 136 mg/L [95–206] (POD 2), and then decreased to 113 mg/L [64–185] (POD 3). The proportion of missing data was 52.7% on POD 4, but surpassed 60% on PODs 5 and 6. In patients who developed an SAE ≥ 3 complication, the median CRP value increased from 92 mg/L [65.5–142.5] (POD 1) to 202.5 mg/L [102.3–282] (POD 3), and ultimately to 182 mg/L [71–276] (POD 5). Six (12%) SAE ≥ 3 complications were diagnosed *and* treated by reoperation *before* POD 3, thus resulting in a deviated course of CRP levels on POD 3 and beyond. Of all SAE ≥ 3 complications, four (8%) were diagnosed *and* treated by reoperation *between* POD 3 and POD 5. In 39 (78%) patients, SAE ≥ 3 complications were diagnosed *after* peak CRP concentration (POD 3), with a median of 8 days^5^–^10^ following CRS-HIPEC. As can be seen in Fig. 1a, in cases of postoperative complications with SAE grade < 3, CRP concentrations peaked on POD 2 (127 mg/L), and also peaked at POD 3 (205 mg/L) in patients with SAE ≥ 3. Median CRP values were significantly higher between patients with SAE ≥ 3 versus patients with SAE < 3 on POD 2 (173.5 mg/L vs. 127 mg/L; *p* = 0.023), POD 3 (202.5 mg/L vs. 104 mg/L; *p* < 0.001), POD 4 (137 mg/L vs. 73.5 mg/L; *p* = 0.002), and POD 5 (182 mg/L vs. 80.5 mg/L; *p* = 0.002).

No similar trends were observed for postoperative WBC levels (Fig. 1b). Overall, median WBC count on POD 1 was 13.4*10^9^/L [10.8–16.2], which declined steadily to 7.9*10^9^/L [5.6–10.7] on POD 5. WBC did not differ significantly on the first 5 PODs between patients who developed SAE grade ≥ 3 complications versus patients who developed SAE grade < 3 complications. However, in 18 patients who developed either anastomotic leakage, bowel ischemia, or perforation, median WBC levels first declined, from 14.7*10^9^/L [11.3–17.8] at POD 1 to 11.0*10^9^/L [9.8–12.9] on POD 3, after which they rose to 12.6*10^9^/L [8.2–16.6] on POD 4 (*p* = 0.031).

### Predictive Value of Biomarkers

**Fig. 2:**
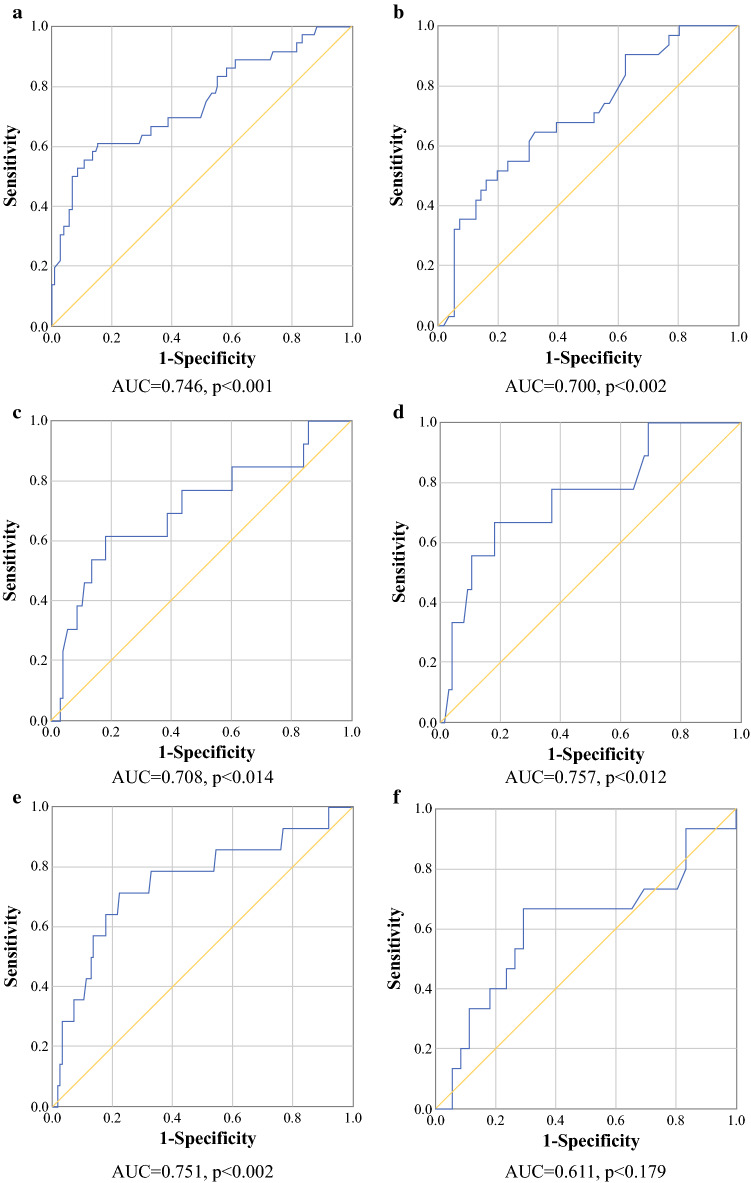
**a**–**f** Receiver operating characteristic curve for C-reactive protein. SAE ≥ 3 at **a** POD 3 and **b** POD4; gastrointestinal complications at **c** POD 3 and **d** POD 4; intra-abdominal abscess at **e** POD 3 and **f** POD 4. *AUC* area under the receiver operating characteristic curve, *SAE* serious adverse event, *POD* postoperative day

Figure 2a, b shows the ROC curves for SAE ≥ 3 against CRP values on PODs 3 (AUC 0.75, 95% confidence interval [CI] 0.65–0.85; *p* < 0.001) and 4 (AUC 0.70, 95% CI 0.59–0.81; *p* = 0.002), respectively. On POD 3, a cut-off CRP value of 166 mg/L had a sensitivity of 61.1% and a specificity of 84.5%. On POD 4, sensitivity and specificity were 54.8% and 76.8%, respectively, for a cut-off CRP value of 116 mg/L. AUCs for the ROC curves for gastrointestinal complications (either anastomotic leakage, bowel ischemia/necrosis, or perforation) against CRP values on PODs 3 and 4 were 0.71 (95% CI 0.54–0.87; *p* = 0.01) and 0.76 (95% CI 0.58–0.93; *p* = 0.01) respectively (Fig. 2c, d). The cut-off CRP value at POD 3 was 188 mg/L, with a sensitivity of 61.5% and specificity of 81.7% (Fig. 2c). At POD 4, CRP ≥ 160.5 mg/L had a sensitivity for a gastrointestinal complication of 66.7% and a specificity of 82.1%. We also examined whether the CRP levels on PODs 3 and 4 can predict an intra-abdominal abscess (Figs. 2e, f). The AUCs for the ROC curves for CRP values on PODs 3 and 4 were 0.75 (95% CI 0.60–0.90; *p* = 0.002) and 0.61 (95% CI 0.44–0.79; *p* = 0.179), respectively. The cut-off CRP level on POD 3 was 166 mg/L, with a sensitivity of 71.4% and specificity of 77.6%.

**Fig. 3:**
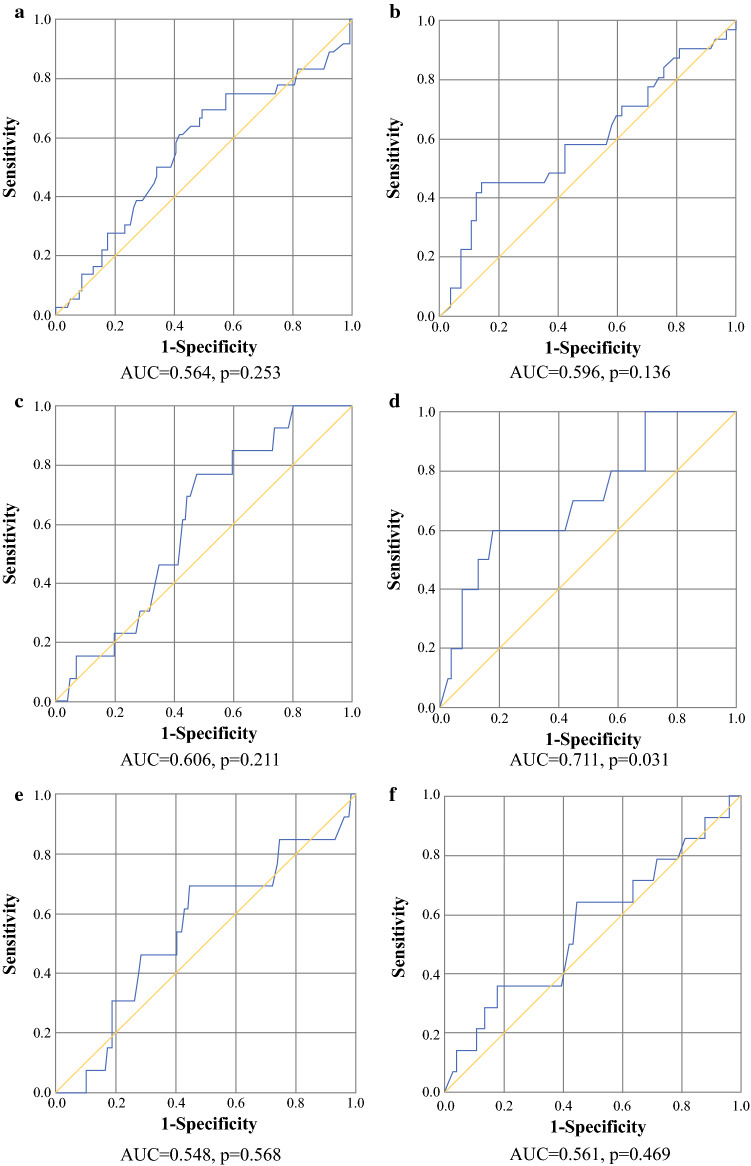
**a–f** Receiver operating characteristic curves for white blood cell count. SAE ≥ 3 at **a** POD 3 and **b** POD 4; gastrointestinal complications at **c** POD 3 and **d** POD 4; intra-abdominal abscess at **e** POD 3 and (**f**) POD 4. *AUC* area under the receiver operating characteristic curve, *SAE* serious adverse event, *POD* postoperative day

Figure 3 demonstrates poor diagnostic accuracy of postoperative WBC levels on POD 3 (AUC 0.56, 95% CI 0.45–0.68; *p* = 0.25) and POD 4 (AUC 0.60, 95% CI 0.47–0.73; *p* = 0.14) in detecting patients with SAE grade ≥ 3 complications (Figs. 3a, b) and intra-abdominal abscesses (Figs. 3e, f). In contrast, the discriminative properties of postoperative WBC levels on POD 4 (AUC 0.71, 95% CI 0.54–0.88, *p* = 0.031) for detecting gastrointestinal complications (either anastomotic leakage, bowel ischemia/necrosis, or perforation) were greater (Figs. 3c, d). In addition, a cut-off WBC level of 11.9*10^9^/L was determined, with a sensitivity and specificity of 60% and 82.1%, respectively.

## Discussion

The aim of this retrospective study was to determine the value of early postoperative inflammatory biomarker levels in identifying patients at risk of developing high-grade SAEs (SAE grade ≥ 3) following CRS-HIPEC. To our knowledge, this is the first study to consider postoperative CRP and WBC levels as a diagnostic tool for identifying high-grade complications following CRS-HIPEC in patients with CRC, appendiceal cancer, and PMP. In cases of SAE grade < 3 complications, CRP concentrations peaked on POD 2, and peaked at POD 3 in patients with SAE ≥ 3 complications. The differences in CRP concentrations were significant from POD 2 until POD 5 between the aforementioned two groups. Most (78%) postoperative SAE ≥ 3 complications were diagnosed after peak CRP concentrations.

In this study, POD 3 and POD 4 were chosen as the time points for calculating AUCs, considering the compelling research^20^ that suggests that postoperative CRP reaches a peak at POD 3 or 4, with better predictive accuracy than CRP on PODs 1 or 2. CRP values on POD 3 had moderate diagnostic accuracy (AUCs > 0.70) for predicting SAE ≥ 3, with cut-off values of 166 mg/L on POD 3 (sensitivity 61.1%; specificity 84.5%). Gans et al.^20^ reported a similar CRP cut-off value of 159 mg/L on POD 3 (sensitivity 77%; specificity 77%) in a meta-analysis regarding ‘major abdominal surgery’. The data observed in our tertiary center suggest that high CRP levels on PODs 3 and 4 indicate a considerable risk for developing high-grade SAEs (i.e. SAE grade ≥ 3). In addition, considering the peak in CRP levels at POD 2 in patients not developing high-grade SAEs versus the peak at POD 3 in patients who do develop high-grade SAEs, clinicians should be extra cautious if CRP levels keep rising after POD 2, especially when they exceed the aforementioned cut-off levels at PODs 3 and 4. These cut-off CRP values may potentially be set as thresholds for additional (abdominal) imaging. External validation should be performed before incorporating this in routine clinical practice.

These aforementioned results suggest that CRP might be utilized to identify patients who are at high risk of developing postoperative SAE ≥ 3 complications. In clinical practice, besides CRP, other variables such as heart rate, temperature, blood pressure, and urinary output are taken into account in decision making for further diagnostics or a reoperation. Taking the inexpensiveness of the laboratory test into account (less than €4 in The Netherlands), also makes this biomarker even more attractive for postoperative monitoring. The current study exclusively analyzed CRP levels and did not consider other clinical parameters. These clinical parameters can influence the pretest probability of developing high-grade SAEs, thereby improving the predictive value of CRP. Hence, if CRP levels are ≥ 166 at POD 3, clinicians might pay better attention to other clinical parameters. Consequently, the early detection rate of high-grade SAEs might increase.

Unfortunately, it was not possible to predict specific complications based on CRP levels alone. Although CRP levels were significantly increased in patients who developed intra-abdominal abscesses or gastrointestinal leakage, the predictive value of CRP for these specific complications was low. This can be explained first by the fact that CRP is a non-specific biomarker and therefore the predictive ability for specific complications is low. Second, the number of events per complication was too small for conclusive statistical analysis; thus, elevated CRP can increase caution for the high possibility that serious complications are evolving, but it cannot precisely predict what complication will develop.

WBC count did not differ significantly between the SAE < 3 and SAE ≥ 3 groups; however, a significant increase in WBC levels from POD 3 to POD 4 was observed in patients with gastrointestinal complications, with a corresponding ROC curve demonstrating moderate diagnostic accuracy. Nonetheless, WBC appears to be less useful, in general, than CRP for detecting high-grade SAEs. A possible explanation might be the low WBC levels due to extensive blood loss and dilution from intravenous fluid administration after CRS-HIPEC. Studies have also shown suppression of the cellular immune response after major surgery, trauma, or injury.^21^,^22^ For CRS-HIPEC procedures specifically, mild leukopenia has been reported as a result of systemic uptake of intraperitoneal chemotherapy.^23^,^24^ These reasons might explain why WBC does not seem to be a reliable predictor of early postoperative complications in patients undergoing CRS-HIPEC. This observation has been previously reported for colorectal surgery.^25^

The current study found significant differences between the SAE < 3 and SAE ≥ 3 groups in relation to sex, number of cholecystectomies, and blood loss. In previously published literature, the effect of sex on postoperative outcomes has been debated. Some earlier studies demonstrated that male patients have a higher risk of complications following colorectal surgery (open and laparoscopic),^26^ and that higher rates of anastomotic leakages were associated with male sex.^27^ However, these aforementioned associations with sex have not been demonstrated in other (retrospective) cohort studies of patients undergoing CRS and HIPEC.^28^–^30^ In the current data, no explanation could be found for this observation. In the SAE ≥ 3 group, significantly more cholecystectomies were performed during CRS. Cholecystectomy has not been earlier described as a risk factor for developing high-grade SAEs after CRS and HIPEC. As most of the cholecystectomies were performed in patients with PMPs, it is very likely that cholecystectomy is an indicator of the extent of disease spread, and thus the extent of surgery. The significant difference in the proportion of cholecystectomies between the SAE < 3 and SAE ≥ 3 groups may be explained via this underlying mechanism. Lastly, median blood loss was significantly higher in the SAE ≥ 3 group. This observation was expected as extensive blood loss has been associated with postoperative morbidity in both general colorectal surgery and the CRS and HIPEC procedures.^26^,^31^,^32^ Perioperative blood loss may, to some extent, reflect the extensiveness of the procedure; there will generally be more blood loss in larger procedures, which results in an increased risk of developing high-grade SAEs.^28^–^30^

### Limitations

There are some limitations to the current study, including, first, the retrospective nature of data collection (including laboratory markers) and the limited study sample size, and, second, the amount of (possibly non-random) missing laboratory data, particularly on POD 4. This is explained by the postoperative HIPEC protocol in the Erasmus MC, which states that laboratory testing should be performed daily on the first, second, and third PODs, and afterwards three times per week on the ward. Third, only the ‘early’ (until POD 5) CRP levels were analyzed in this study, since most CRP values after POD 5 were more likely to be ‘missing not at random’: missingness related to a speedy recovery, and thus unnecessary laboratory testing and/or hospital discharge (‘confounding by indication’). However, this observation was not considered an issue for this particular study, considering its aim was to evaluate CRP as a biomarker for *early* detection of SAE ≥ 3 complications. In addition, Medina Fernandez et al.^16^ suggested that CRP cut-off values might only be of value in the first postoperative week, as their results found CRP levels in the second postoperative week to be not significantly different between patients who developed infectious complications and those who did not, following CRS-HIPEC for ovarian PC.

## Conclusion

With a cut-off value of 166 mg/L on POD 3 after CRS-HIPEC, CRP is a good screening test with high specificity in differentiating between SAE < 3 and SAE ≥ 3 complications. Following CRS-HIPEC, postoperative CRP levels might not only aid in patient selection to prevent overuse of imaging but also for earlier and safe hospital discharge. More prospective studies are needed to more accurately determine the predictive ability of early postoperative CRP levels, in combination with clinical parameters, after CRS-HIPEC.
